# How do cancer patients refuse treatment? A grounded theory study

**DOI:** 10.1186/s12904-023-01132-5

**Published:** 2023-02-07

**Authors:** Hamid Reza Khankeh, Reza Vojdani, Mahboobeh Saber, Mohamadhadi Imanieh

**Affiliations:** 1 Health in Emergency and Disaster Research Center, University of Social Welfare and Rehabilitation Sciences, Tehran, Iran; 2 Department of Clinical Science and Education, Karolinska Institute, Stockholm, Sweden; 3 Hematology Research Center, Shiraz University of Medical Sciences, Shiraz, Iran; 4 Department of Medical Ethics, School of Medicine, Shiraz University of Medical Sciences, Shiraz, Iran; 5 Gastroenterohepatology Research Center, Shiraz University of Medical Sciences, Shiraz, Iran

**Keywords:** Grounded theory, Qualitative research, Treatment refusal, Neoplasm, Resilience

## Abstract

**Background:**

All cancer patients, except for a small fraction, seek treatment after becoming aware of the disease. That small fraction do not seek any treatment due to various reasons, and this phenomenon is unknown to us. Therefore, the present study aimed to discover the reasons for treatment refusal in cancer patients.

**Methods:**

This qualitative grounded theory study was conducted on 22 participants including patients, caregivers, physicians, and nurses. Purposive theoretical sampling was employed. Data were collected through in-depth interviews. All interviews were gradually transcribed and analyzed. Data analysis was carried out through the three-step method of open, axial, and selective coding and was continued until theoretical saturation. Straussian Grounded Theory was used for data analysis.

**Results:**

A total of 4 themes and 20 sub-themes were extracted in this study. The core variable extracted from the interviews was “resilience” Other related themes included encounter with cancer, fighting cancer, and coping with cancer. The findings showed that in the context of fighting cancer, patients lost their resilience through various processes and refused treatment.

**Conclusion:**

Cancer patients abandon the treatment in silence, oncologists and even family members being unaware of the matter. In other words, refusal of treatment is like an iceberg and the majority of the patients who have abandoned treatment are unknown to the health system. The model obtained in this study can increase the knowledge of the process that leads patients to lose their resilience against cancer and abandon treatment, which can increase the possibility of recognizing and predicting treatment refusal for oncologists.

**Supplementary Information:**

The online version contains supplementary material available at 10.1186/s12904-023-01132-5.

## Background

After being diagnosed with cancer, most people visit oncologists and follow the diagnostic and therapeutic recommendations with sensitivity and caution regardless of the type of cancer, disease stage, and age [1]. However, some patients reject a part or the entirety of diagnostic and therapeutic recommendations [2]. Considering the invasive nature of the disease, delays in starting or abandoning the treatment would have an obvious impact on the quality and quantity of the patients’ lives [3–5].

Patients’ adherence to treatment recommendations can be described in the form of a spectrum. One side of this spectrum is complete adherence and the other side is the complete rejection of treatment recommendations including surgery, chemotherapy, and radiotherapy. The phenomenon of treatment refusal involves the rejection of the main treatment programs recommended by physicians and does not include a relative lack of cooperation that would not impact the main treatment process or the outcome [6, 7].

Up to now, mostly quantitative-analytical studies have been done in this area, which have provided the results of the investigations of the relationships between different factors based on cancer databases. According to the findings, increase in age, low level of education, low income, cancer stage, low weight, poor performance state, presence of other catastrophic illnesses, and lack of social support are effective in increasing the probability of treatment abandonment in cancer patients [4, 5, 8–10].

However, other studies have indicated that the experiences and beliefs of patients can greatly influence their acceptance and cooperation or abandoning of treatment. Patients make decisions regarding the refusal or continuation of treatment based on their personal values [11]. In fact, the benefits and harms of the suggested treatments, as the basis for physicians’ treatment recommendations, do not matter as much to patients and they make their decisions based on their own experiences, preferences, and values. This even leads to the refusal of curative treatments by some patients. Other studies have also emphasized the importance of patients’ beliefs in treatment abandonment [11, 12]. In one study on the experiences of patients with lung cancer who had abandoned treatment, the importance of self-efficacy, faith, and acceptance of fate was highlighted. Lack of trust in the health system was another point raised by these patients through the expression of negative experiences and requests for detailed information accompanied by complaints of having received inadequate information. Another factor mentioned by the patients was the lack of willingness to undergo the pain and suffering resulting from treatment procedures [13]. These experiences and beliefs were so influential in the patients’ decision-making that despite receiving adequate information about the methods and effectiveness of the treatments, they still refused to accept them [14]. In the study by White, the patients who had abandoned treatment were those who believed that conventional treatments would have negative effects on their quality of life and that they had to seek healing in their own mind and soul. Hence, they replaced the conventional treatment with complementary medicine. Negative initial experiences and unsympathetic and strict attitudes of oncologists have also been strongly influential in patients’ decision for rejecting diagnostic-therapeutic interventions [15]. Moreover, death of close ones due to cancer after conventional treatments is a difficult experience, which often affects the choice of treatment and the decision to abandon conventional treatments [16]. One of the limitations of the above-mentioned studies was that the participants were selected among the patients with the same type of cancer including prostate, breast, and lung cancers.

As mentioned earlier, mostly quantitative-analytical studies have been conducted on treatment refusal in cancer patients. In addition, only a limited number of studies have focused on the mental aspects of this phenomenon and they have been carried out on participants with the same type of cancer. On the other hand, the phenomenon of treatment refusal is a culture-bound, complex, experience-based, and individual concept. Thus, it is essential to conduct studies in order to reach an understanding of the culture in this area. The current study has been carried out with a qualitative grounded theory design in order to reach an explanation of the process of treatment abandonment in cancer patients, as an occurring phenomenon.

## Methods

This study was designed based on the grounded theory, as proposed by Strauss and Corbin [17] . Grounded theory is a suitable methodology for taking a fresh look at a familiar subject or attempting to discover a phenomenon that has not been properly described. In this method, the perceptions and experiences of individuals are explored in the common social structure.

### Participants

The study participants included patients, caregivers, and healthcare service providers who had deep, direct experiences of interacting with cancer patients who had abandoned treatment. The patients participating in the study were individuals with cancer who, while being aware of the disease, had abandoned the conventional treatment either temporarily or permanently for over six months at certain stages including surgery, radiotherapy, and chemotherapy and had refused to accept their physicians’ diagnostic-therapeutic suggestions. The exclusion criteria were limited to unwillingness to participate and not meeting the minimum physical and mental requirements for interview. For caregivers and healthcare service providers, the inclusion criteria were having experiences of direct contacts with cancer patients who had refused treatment and being willing to cooperate. Overall, there were 22 participants in this study including twelve patients, five caregivers, and five healthcare service providers, two of whom were oncologists. A demographic presentation of participants are illustrated in Table 1.

**Table 1:** Demographic presentation of the participants (*n* = 22) and role of them

Gender	
Female	15
Male	7
Age	
Mean	49.2
Min-Max	22–82 years
Role	
Patient	12
Care giver	5
Oncologist	2
Nurses	3
Cancer diagnosis (*n* = 12)	
Breast cancer	4
Liver cancer	1
Esophagus cancer	1
Lung cancer	1
Abdominal cancer	1
Chondrosarcoma	1
Uterus cancer	1
Bowel cancer	2

### Data collection

The data were collected through in-depth interviews from March 2016 to October 2017. The time and place of the interviews were arranged by the participants. The interviews were carried out by the correspond researcher who was an expert in data collection through interview. The interviews lasted 25–70 minutes. In some cases, the physical conditions of the patients were influential in limiting the interview duration. During interviews with the patients and caregivers, the observations were documented through field notes. Regarding the procedure, the researchers first recorded the contents of their own contemplation and internal dialogue regarding their observations in an orderly fashion in form of a memo. The participants first faced the following question: “Tell us the story of your disease. How did you get to the point of abandoning the cancer treatment?” Then, probing questions were posed based on the understandings, experiences, and emotional reactions of the participants. All participants were informed about if there is the possibility of getting in touch with them in case there were any other questions.

Initially, the participants were selected through purposive sampling. As the study progressed, the importance to clarify the ambiguous dimensions of some concepts and the selection of participants continued through the theoretical sampling. To highlight this issue, the use of complementary medicine and abandoning the conventional treatment were amongst these cases. Thus, selection of participants was executed on this ground, and by interviewing the aforementioned participants, it provided a more accurate description of this concept for the researchers. The non-profit organizations and their role in caring for and supporting patients as well as their families was another concept, which was selected to clarify the dimensions of the issue. Theoretical sampling continued until the end of the study to reach the conceptual model.

### Data analysis

Data collection and analysis were carried out simultaneously and parallelly using the approach proposed by Corbin and Strauss. In doing so, all the interviews were recorded and transcribed. Data analysis was performed through the three steps of open, axial, and selective coding and was continued until theoretical saturation. The interviews were analyzed one by one and gradually. In the open coding stage, the interview transcripts were repeatedly reviewed. The meaning units and subsequently the codes were extracted. The two main strategies of questioning the data and constant comparison were adopted. In this stage, memos and field notes were helpful in the proper understanding of the meaning units. In the axial coding stage, the categories were formed through the process of relating codes to each other in order to group the similar codes with each other and reduce the number of categories. Moreover, a search was carried out to discover the relationships between categories through the probing questions and constant comparison. Next, the categories were merged and the axial categories were formed. Finally, selective coding was carried out for all categories and subcategories using the constant comparison and focus group strategies. The focus group session was held with the presence of hematologist oncologists. The relationships between the concepts and the categories were presented and a number of questions were posed in order to complete the relationships between the concepts, mechanisms of effect, and effect sizes. At this stage, the core variable was discovered. Then, the existing processes were extracted and the conceptual model was formed with the completion of the relationships. Theoretical saturation was achieved at this stage.

### Trustworthiness

In this study, the four criteria proposed by Lincoln and Guba were used to ensure trustworthiness. The researcher spent over a year collecting and analyzing the data, and this prolonged engagement with the data and field notes paved the way for credibility. In the process of data collection, the verification of peers and participants was utilized. In addition, the focus group session that was held provided the opportunity to verify the concepts and the relationships between them and helped meet dependability. After obtaining the initial codes and concepts, the literature in the field was used to ensure confirmability. Finally, the researcher provided a detailed description of all the involved factors including the participants, data collection and analysis methods, and study limitations, hence ensuring the transferability of the data.

## Results

In this study, the decision-making process involved in treatment refusal amongst cancer patients was explored through the grounded theory methodology. A total of 530 initial codes were extracted, which revealed four categories and twenty subcategories in the process of data analysis.

**Fig. 1:**
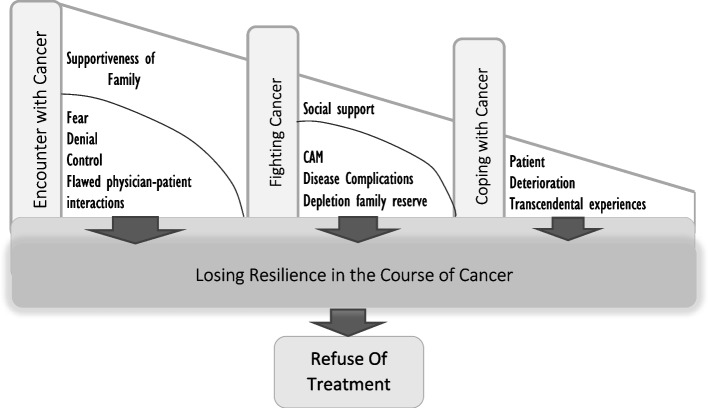
Cancer patient refuse of treatment model

Figure 1 illustrates how the four categories are related to each other. “Losing resilience against cancer” is the core variable at the center of this model, and the three other categories are related to this concept one by one. The process of abandoning treatment by cancer patients transpires in different ways, and before stopping the treatment, patients experience lack of resilience and endurance.

The category “Encounter with cancer” explains part of abandoning treatment, which is at the time of being diagnosed with cancer, and explores the contributing factors. Denial, fear, flawed physician patient relationship, shock of encounter can trigger a process that can lead to lack of resilience and endurance that ultimately leads to abandoning the treatment.

The “fighting cancer” category includes the description of the contributing factors leading to the refusal of treatment, in which the patient is utterly fighting the disease. Treatment complication-disease complication, suffering from the disease, shortcoming in sustainable support, can explain the conditions that patient’s experience, which leads to the patient’s lack of endurance and discontinuation of their treatment.

The category “coping with cancer” elucidates the patient’s resilience and endurance under the circumstance that the cancer has progressed. Disease progression, depletion of the patient can lead to lack of endurance by ultimately abandoning the treatment. These categories are summarized in Table 2.

**Table 2:** Cancer Patient Refuse of Treatment: Category and Sub Category

Category	Sub Category
**Encounter with Cancer**	Shock of encounter
	Interaction of the treatment team with the patient
	Flawed physician-patient interactions
	Denial of the disease
	Incorrect understanding of the disease
	Fear
	Supportiveness (family-patient)
	Control
**Fighting Cancer**	Disease exacerbation, end of denial
	Complementary therapies as alternative treatment
	Treatment complications, disease complications
	Treatment facilitators
	Suffering of the disease
	Shortcoming in sustainable support
	Depletion of family reserves
**Coping with Cancer**	Deterioration of the patient
	Transcendental experiences of the patient
	Disease progression, necessary care provision
	Regrets due to delaying the treatment
**Losing Resilience in the Course of Cancer**	Refusal of treatment
	Norm violation
	Depression

### Encounter with cancer

All cancer patients remember the time they became aware of their disease. The encounter with cancer affects all individual and social dimensions of a person’s life. Different dimensions of this category included the shock of the encounter, care provided by the treatment team, flaws in patient-physician interactions, denial of the disease, fear of the disease, incorrect and flawed understanding of the disease, familial support, and search for sympathetic care.

The results indicated that experiencing the shock of an encounter with cancer is a common and challenging event. Physicians and nurses often witness this stage of people’s encounter with their disease. In this stage, the sympathetic care and support provided by patients’ close ones and the treatment team are strong determinants.

For the majority of patients and their families, the stage of encounter with the disease is often passed with difficulty. When encountering the disease, a treatment team is often with the patient. Physicians make the patient aware of the situation ahead by expressing the conditions of the disease and treatment recommendations and try to prepare the patient for disease acceptance by gradually presenting the information. This is an unspoken agreement between all members of the treatment team, namely physicians and nurses, that they do not state all the facts related to the disease at the early stages and refer only to a part of the information in order to direct the patient toward the implementation of the treatment protocols. A concerted effort is also made to give hope to the patient and show the promising aspects of the disease in a coordinated manner.

“*In reality, we push the patients, we deceive them so that they’ll come. First we tell them to attend three sessions of chemotherapy. Then we say two more; let’s see how it goes. Then we tell them that it is working well and ask them to come for two more sessions. Then, we tell them to come for one more session just to make sure. We do not tell the patients that they should come for eight sessions from the beginning. Even though the treatment protocol is eight sessions from the start, we deceive the patients, so that they can tolerate chemotherapy; otherwise, chemotherapy would be really difficult*...” (P1- nurse).

Cancer is a serious, long-lasting disease with numerous complications. Under such circumstances, cooperation between the doctor and the patient is only possible through mutual trust, which is formed through proper interaction between the patient and the oncologist. In cases where this interaction is not well achieved, it will have negative impacts on the patient’s decision.

“*Unfortunately, the doctor doesn’t provide proper explanation to patients. If the doctor had told me that this disease was dangerous and I had to undergo surgery, I would not have given up the surgery. If you are literate, the doctor will answer your questions. For me, however, he just told me to go and do this; he did not explain. So, I said to myself that I just had to let it go; I didn’t think how the situation would be.*..” (P3- patient).

Physician-patient interaction includes the way the news of cancer is delivered, explanations regarding diagnostic and therapeutic interventions, and patient prognosis. To express these matters compassionately and gradually, one must act with sensitivity.

“*I went for biopsy. When I took the results to my surgeon, the doctor came to visit me with his assistants. Then, they told me very abruptly and clearly that my leg had to be amputated! At that moment, I felt so horrified. I wasn’t willing to do what he had said under any circumstances.*..” (P4- patient).

In such a situation, patients experience a level of anxiety that is more than they can endure. This led to the discontinuation of treatment for more than a year among the patients participating in the present study. In fact, the physicians’ inappropriate interactions with the patients created a level of pain and suffering that was beyond their tolerance. Hence, they had abandoned the treatment for more than a year and refused to accept the treatment recommendations due to intolerance.

Denial is one of the most common reactions among the people facing unpleasant situations. The transience of denial puts it at the level of an adaptive reaction. According to the results of the current study, the long-term denial of disease is considered an influential factor in treatment abandonment. This is intensified by limited clinical symptoms, hiding the disease from others, and insisting on the pre-disease lifestyle.

“*During my sister’s illness, we had travel plans, family gatherings, parties, shopping... It was good that we did not need to go to the hospital and see other patients. My sister and I were just satisfied that her schedule during the illness was the same as before*...” (P5- caregiver).

The participants who had refused to start the conventional treatment due to denial or had abandoned it in the early stages insisted on maintaining a pre-disease lifestyle. Another dimension of disease denial was hiding the disease from others. In this scenario, cancer patients hide the disease from their family members, friends, and even family physicians and try to make everyone think of them as healthy, so that they can have the same routine life as a healthy person. These cases were seen among the current study participants in situations where the clinical signs of cancer were limited or after the first stage of chemotherapy when the symptoms disappeared.

“*My illness started with coughs. They said I had lung cancer and I got chemotherapy for eight months. Then, the doctor referred me for surgery, but I did not go. The doctor said I had a tumor and I thought to myself he was just saying that; I was fine. After all, I had been receiving chemotherapy for months. I told my children that the doctor had said I was fine, and I was fine indeed. I was living a normal life; I had completely forgotten about the cancer. I exercised, walked, but after a year, I started coughing again and I felt what the doctor had said might be true*...” (P3- patient).

The results of this study showed that some patients faced cancer with presumptions. As a result, the decisions about treatment were made before interacting with an oncologist and under the impact of those presumptions. In such cases, the patient resists diagnostic and therapeutic interventions without knowing the exact type of cancer, the degree of invasiveness, and the stage of the disease and refuses to cooperate. One of the oncologists described his interaction with a patient who did not accept the proposed treatment as follows:

“*The patient had Hodgkin disease, but she said she did not want to be treated and that she would fight the disease herself... I talked to them patient and her wife for half an hour. I told him that she could be treated and the disease was curable at that time, but if she wasn’t treated, it would progress and destroy her. No matter how much I explained, she did not accept and refused to allow me to start the treatment for her*...” (P2- oncologist).

Physicians try to portray an accurate and logical picture of the disease in patients’ minds, but the picture of cancer is influenced by patients’ life experiences. Therefore, despite physicians’ explanations, some patients may never accept the treatment recommendations.

### Fighting cancer

Over time, the disease progresses and starts to reveal its serious nature. The signs and symptoms increase, and the patient actually experiences the pain and suffering of cancer that was the cause of fear in the previous stage. In this situation, the patient may not be able to endure this excruciating pain for various reasons and leave the treatment incomplete. Intolerance in this situation occurs for several reasons. One of the reasons patients abandon treatment is the complications of treatment. At this stage, patients sometimes refuse to accept the conventional treatment because of complementary treatments. Chemotherapy, radiotherapy, and surgery can cause numerous complications. Despite the transient nature of the majority of these complications, if the patient does not have the capacity to tolerate the situation, they will reject the treatment and refuse the treatment recommendations.

“*I had a patient who had lymphoma. She had undergone chemotherapy twice and needed to go for a third session. However, she wouldn’t accept, saying how many times a woman can lose her hair!*” (P2- oncologist).

“*After biopsy, the doctor told me that I had to undergo chemotherapy and that my leg had to be amputated. I didn’t want to do it, I couldn’t. My hair was very long and I couldn’t bear to lose it. I could not imagine life without legs and I couldn’t go on. I wanted to go to college, so I abandoned treatment. I said let the chips fall where they may*” (P4- patient).

In the process of cancer treatment, patients are resistant to all types of surgery, especially if they cause an apparent defect. Chemotherapy is also painful due to its many physical complications, toxicity, and hair loss. These therapeutic interventions cause great sufferings for patients and if they do not have sufficient capacity to endure, they may abandon the treatment at this stage.

According to the results of the present study, patients seek complementary medicine due to their concerns about the complications of chemotherapy and surgery and in order to avoid the complications of conventional treatment. In some patients, unsuccessful experiences with conventional treatment in their loved ones is a factor influencing the use of complementary medicine. Combining complementary medicine with spiritual beliefs by some providers of these therapies is another reason why patients pay attention to them.

Based on the results of this study, there are interventions in the treatment process that, without interference with the conventional treatment, reduce the damage and suffering of cancer while increasing tolerance against the suffering of treatment. This is how one of the participating patients described his experience in this area:

“*I was scared and hated having a crooked line on my chest. So, I said that I would not have the surgery. My brother who was in Canada told me not to refuse the surgery. He told me that I was young and I could ask them to give me a prosthesis, so that my appearance would not change. He told me that I could even get implants for both of my breasts*” (P11- patient).

Interventions of this kind would reduce the suffering of the disease and increase the probability of treatment acceptance and resilience in patients.

A person’s illness affects the entire lifestyle of their family members. The support of a family for a sick member means changing the family priorities, and family members spend their emotional and financial resources to meet the patient’s treatment needs and improve the patient’s quality of life. Given the importance of compassion in the Iranian culture, family members sometimes include a large group of people with even distant familial relationships, all of whom strive to improve the patient’s condition. Nonetheless, prolonged illnesses can deplete people’s emotional reserves and affect the process of disease management.

“*In the first stages of treatment and hospitalization, the patient comes with one’s family. The family insists on the treatment process being carried out in the best way possible. As time goes by, however, the family’s reserves gradually run out. It gets to the point where the family wants to do something, but is no longer able to*. “ (P1- nurse).

Inadequate emotional reserves of a family can cause the emotional ties to be challenged by the difficulty of a family member’s long-term illness. This crisis affects patients and targets their resilience, thereby affecting the decision to continue or abandon the treatment.

### Coping with cancer

Leaving aside the fraction of cancer patients who are cured, other patients, after periods of relapse/recurrence, reach a point where the disease dominates their bodies and they are practically subdued by the disease. The patients who have abandoned conventional treatment during the stages of encountering and fighting cancer and whose disease has hence progressed faster experience regret due to their previous decisions. According to the results of this study, some patients refuse to receive any kind of treatment after being physically depleted due to the progression of the disease. At this stage, doctors recommend chemotherapy or radiotherapy to reduce the symptoms or prescribe medications that improve the patients’ physical conditions. However, due to the reduced physical capacity and dependence on others for their daily needs, patients may have no desire to receive any treatments even to improve their general health.

Many patients have transcendental experiences in this situation. According to current study results, some patients in these physical conditions have transcendental experiences in dreams or even experiences such as seeing their dead relatives in a state between wakefulness and sleep. The effect of such spiritual experiences on patients in these conditions is an easier acceptance of death and even attempts to hasten it by not taking the medications.

### Losing resilience

This concept was identified as the core variable in this study, which was associated with the aforementioned three main categories and their subcategories. Traces of this concept could be observed in all the sentences the patients used to describe their suffering and despair in the course of the disease.

“*I’ve had a headache for a week and I’ve not been able to see for a few days. I have double vision (cries slowly...). I won’t even go to the doctor anymore, I want to let go, I don’t want chemo anymore... I don’t want to wait and see where it will hit me. What would happen to me? This is harder, it’s very difficult*...” (P16- patient).

There comes a time when the patient cannot tolerate the disease anymore. Numerous reasons such as disease symptoms, treatment complications, and fear lead the patient to this point. Each patient describes this condition in a different way. However, all patients who have abandoned treatment have experienced it.

Cancer has a great impact on the patient’s lifestyle from the beginning and the course of the disease is affected by various processes and actions. The treatment refusal process in cancer patients shows how a patient experiences the contexts of encountering cancer, fighting cancer, and coping with cancer during the illness and how the factors in each context cause the patient to lose resilience, which is equivalent to the refusal of physicians’ treatment recommendations.

## Discussion

A minority of cancer patients do not accept their physicians’ treatment recommendations. The results of the present study indicated that a wide variety of reasons were influential in this type of decision. This study aimed to explore the phenomenon of treatment refusal in cancer patients. Interpreting the themes and categories in this area provided a deeper understanding of the phenomenon. In this study, resilience, as a core variable, was directly and indirectly related to the other categories including encounter with cancer, fighting cancer, and accepting cancer.

According to the results, patient’s first experiences dealing with cancer is a difficult one. Being exposed to trauma describes the emotional experience of patients, which is recognized by the treatment team and has been described in other studies. The first thing that crosses the patients mind is that cancer is an incurable disease, to the extent that they say cancer it is like a death sentence [18]. In such a circumstances, the caring role of physicians and nurses is vital. In this study, providing information on need to know bases by highlighting the positive and less difficult aspect of the impending procedures were the strategies, which was utilized by the treatment team in order to provide compassionate care. This approach is in line with the methods described by the physicians and nurses. Empathetic approach by the physician, which means he/she understands the patient’s fear, by creating hope-based communication can often lead to making better choices during the treatment process. In this study, two-way-interaction and spending suitable amount of time to gain the patients’ trust was imperative [14]. Other studies have also considered appropriate communication between the physician and the patient, separating the process of cancer diagnosis from treatment planning, and facilitating the patient’s access to the treatment team as effective factors in the adherence and acceptance of treatment suggestions, which were in line with the results of this study [18] The results of Sharf’s study also indicated that improving doctor-patient interaction is very vital, when attempting to deliver the bad news. Through in-depth communication, the physician can gain better understanding of the reasons as to why the patients resist the recommendations; hence, he/she will have a better chance convincing the patient to accept the treatment options [13].

The results of the present study showed that denial at the initial stage and after the first stage of chemotherapy, when the apparent symptoms of the disease disappear, was one of the reasons for treatment abandonment. In addition, a prolonged denial phase increased the likelihood of refusing the treatment recommendations. The prevalence of denial in cancer patients has been studied, and up to half of patients experienced denial at some points in the course of the disease. However, these studies have not assessed the relationship between denial and adherence to treatment [19].

The complications of cancer treatment were also among the reasons for treatment refusal mentioned by the patients in the current research. Various studies have similarly referred to treatment complications as one of the reasons why patients seek complementary medicine [14, 20–22]. The results of the present study showed that at the stage of encountering cancer, family support was an important factor in the endurance of cancer patients. Generally, family members’ support is influential in adherence to medical care. An individual’s children, spouse, parents, siblings, and sometimes more distant relatives form a support circle. In the Iranian culture, patients’ family members take care of them by attending medical centers and encourage them in various ways to continue the treatment. In this way, supportive families play their role in improving the patient’s resilience. Kreling also disclosed that supportive family members encouraged their patients and followed up their treatment, while the patients who did not have familial support were more likely to reject the treatment recommendations.

Past experience of a loved one with cancer, particularly their death following conventional treatment, was another factor increasing the likelihood of treatment refusal in the present research. This experience creates deep-seated fear and anxiety in patients and impairs their tolerance against cancer. In the study by Van Kleffens, the discontinuation of treatment was predictable in the patients who had experienced the death of their relatives or close friends due to cancer, and there was an increased possibility of seeking complementary medicine after abandoning the conventional treatment [23]. In the same line, the findings of the study by Goldberg revealed a higher prevalence of treatment abandonment in the cancer patients who had lost a loved one due to cancer [24, 25].

Misconceptions about the nature and course of cancer and the complexity of understanding the concept of uncertain treatment outcomes at different stages were among the reasons for treatment refusal. Various studies have shown that in situations where the patient does not have a correct understanding of the concept of uncertainty, physicians’ treatment recommendations that raise the possibility of recovery or recurrence increase the rate of treatment refusal amongst patients [7, 26].

As time goes by and cancer progresses, patients are less likely to accept their physicians’ treatment recommendations due to physical depletion. In this situation, the complications of the disease and treatment limit patients’ capacities. The results of the current study demonstrated that the patients’ deterministic beliefs at this stage of the disease were influential in the rejection of treatment proposals. The study by Yunesi aimed to evaluate the adoption of deterministic perspectives by cancer patients in order to adapt to the course of the disease [27]. However, no association was observed between the patients’ beliefs in this philosophical viewpoint and treatment refusal.

The current study provided a deeper understanding of the factors and conditions, by which cancer patients lose their resilience and reject the proposed treatments. In the meantime, such factors as social support were also mentioned to increase the patients’ resilience. Zhang conducted a research in 2017 to investigate resilience and quality of life in patients with breast cancer [28]. They pointed to the role of social support in the improvement of resilience and quality of life among the patients.

The majority of studies on the resilience of cancer patients have focused on interventions such as group therapy, positive psychology, and behavior therapy or mindfulness and have not addressed how patients pass the course of cancer [27, 28]. In addition to the aforementioned studies, a conceptual model was designed to describe the resilience in cancer patients. In this model, individual and environmental characteristics, cancer-related events, the individual reactions and adaptation are depicted in a cycle for recalibration. This can change the patient’s condition from distress to resilience, and alternatively, it refers to interventions as an effective factor in this cycle [29]. In this model, compliance to treatment or its refusal is not mentioned, because the patient progress was desired and objectified. The current study, however, discussed the relationship between the patients’ experiences and conditions at the time of cancer and various processes in the course of the disease that influenced their resilience, and proposed a suitable model.

The strength of this study was that it provided the opportunity to identify the phenomenon of treatment refusal in cancer patients without any limitations regarding the type of cancer and age and outlined the relationship between this phenomenon and resilience among cancer patients. Although only a limited number of cancer patients abandon medical treatments, there are several reasons influencing the decisions of this limited group. Providing a model to explain the process of treatment refusal allows for a systematic look at this variety of reasons.

The limitation of this study was that the duration of the interviews was limited due to the patients’ physical conditions. In addition, it was not possible to conduct the second interview with some patients due to disease progression and adverse physical conditions, which might have affected the results.

## Conclusion

The model presented in this paper for treatment refusal in cancer patients can enhance our understanding of the process that leads patients to the point of losing their resilience against cancer and abandoning treatment. This study touch upon some considerations for physicians to promote their interactions with patients who decide not to continue the treatment recommendations. In the course of cancer, certain factors that lead to lack of resilience are important. During the early stages of cancer, proper interaction between the physician and patient is the key. At times, the bad news is delivered by the physician during the first meetings. The disjunction between the treatment team when delivering the bad news, and implementation of different treatment strategies is something that has to be reassessed. Follow-up and care for patients with prolonged denial is highly recommended by considering the important role of supportive families and to improve patient’s resilience. Hence, it is crucial to strengthen the role their families to increase treatment acceptance. Furthermore, it is vital to develop and expand the role of palliative care, and to start it from the early stages of cancer. Finally, by identifying the phenomenon of treatment refusal, it can lead to improved patient resilience, while considering patient’s choice when providing care and treatment.

Individuals who have had a loved one dying due to cancer following conventional treatment, people who deny their illness for a long period, and people who do not trust the medical structure or are interested in traditional treatments are among the groups that need to be understood in order to provide the possibility of a deeper interaction with the treatment team towards reducing the likelihood of treatment refusal. This detailed understanding of the refusal process will be extremely helpful in helping cancer patients.

## Supplementary Information

**Additional file 1.** Interview Guide.

## Data Availability

The datasets used and/or analysed during the current study are available from the corresponding author on reasonable request.
