# Supplementary material for: How do cancer patients refuse treatment? A grounded theory study
Source: BMC Palliat Care. 2023 Feb 7;22:10. doi: 10.1186/s12904-023-01132-5 (PMC9903566; doi:10.1186/s12904-023-01132-5)
Supplement: Supplementary file 1 — Additional file 1. Interview Guide. [file 12904_2023_1132_MOESM1_ESM.docx]

| Interview guide |
| --- |
| **Patient and care giver Interview** |
| Tell me the story of your illness  How did you feel when you found out you had cancer?  How did you decide to abandonment cancer treatment?  What were the conditions that made you refuse the suggested treatments?  How was chemotherapy and radiotherapy?  How do you see the side effects of treatment?  What did the treatment of the disease mean to you?  What conditions made the situation more difficult or easier?  Explain how your family is dealing with your illness?  Explain a day with illness?  *In cases where more explanations were needed, the following questions were used.*  Please explain this item that you mentioned more.  How did you feel in that situation?  *And finally*  Is there anything else you want to talk about? |
| **Health care provider interview** |
| How do patients face their illness?  How do people experience the beginning of the disease?  How do patients refuse treatment?  How was the process of refusing treatment experienced by the patient?  What were the patients' reactions to different chemotherapy, radiation and surgery treatments? |
